# Mechanistic Aspects of Apiaceae Family Spices in Ameliorating Alzheimer’s Disease

**DOI:** 10.3390/antiox10101571

**Published:** 2021-10-02

**Authors:** Niti Sharma, Mario A. Tan, Seong Soo A. An

**Affiliations:** 1 Department of Bionano Technology, Gachon University, 1342 Seongnam-daero, Sujeong-Gu, Seongnam 461-701, Korea; nitisharma@gachon.ac.kr; 2 College of Science and Research Center for the Natural and Applied Sciences, University of Santo Tomas, Manila 1015, Philippines; matan@ust.edu.ph

**Keywords:** Alzheimer’s disease, amyloid-*beta*, antioxidant, enzyme inhibitors, essential oils, natural products, neuroprotective, spices

## Abstract

Alzheimer’s disease (AD) is one of the most prevalent neurodegenerative diseases worldwide. In an effort to search for new strategies for treating AD, natural products have become candidates of choice. Plants are a rich source of bioactive and effective compounds used in treating numerous diseases. Various plant extracts are known to display neuroprotective activities by targeting different pathophysiological pathways in association with the diseases, such as inhibiting enzymes responsible for degrading neurotransmitters, reducing oxidative stress, neuroprotection, inhibiting amyloid plaque formation, and replenishing mitochondrial function. This review presented a comprehensive evaluation of the available scientific literature (in vivo, in vitro, and in silico) on the neuroprotective mechanisms displayed by the extracts/bioactive compounds from spices belonging to the Apiaceae family in ameliorating AD.

## 1. Introduction

Neurodegenerative disease is a broad term used to describe a range of conditions primarily affecting neurons, ultimately leading to the progressive loss of normal motor and cognitive functions. According to a recent report, around 50 million people suffer from such diseases worldwide [1]. Alzheimer’s disease (AD) is one of the most prevalent neurodegenerative diseases that affect millions throughout the world. US Food and Drug Administration (FDA)-approved drugs for ameliorating AD symptoms include acetylcholinesterase inhibitors (rivastigmine, galantamine, tacrine, donepezil) and the NMDA receptor antagonist (memantine) [2,3]. A new drug, Aduhelm (antiamyloid antibody intravenous (IV) infusion therapy), was recently approved by the FDA, which can delay clinical degeneration, with benefits to both cognitive and motor functions in AD patients. However, all these treatments suffer from side effects ranging from headache, nausea, confusion, dizziness, fall, and amyloid-related imaging abnormalities (ARIAs, such as swelling in the brain and microhemorrhages/superficial siderosis). Therefore, it is of utmost importance to search for new strategies and drugs that can help delay the onset and progression of the disease. AD hallmarks include alterations in the levels of neurotransmitters, amyloid-*beta* (Aβ) plaque deposition, and atypical tau protein phosphorylation [4]. The vital role of oxidative stress in neurodegenerative disorders is also well recognized. The neurons are more susceptible to free-radical-mediated damage compared to the rest of the body [5]. Under normal physiological conditions, antioxidant enzymes help overcome the oxidative stress generated in the body, but in AD, these enzymes are unable to achieve this task [6]. Due to the accumulation of Aβ, the level of reactive oxygen species (ROS) increases, which severely affects the function of various proteins, enzymes, transporters, and ion channels due to oxidative damage [7,8]. Superoxide radicals also interact with nitric oxide produced by activated microglia, enhancing peroxynitrite and other RNS formation [9,10]. The ROS/RNS build-up with the activation of apoptosis and reduction of antioxidant enzymes has a catastrophic effect on cholinergic regions involved in cognitive performance [11]. Thus, controlling ROS levels can be an alternative approach in the pathophysiology of AD. Numerous reports suggest the protective role of phytochemicals against oxidative stress and neuroinflammation, which are the key hallmarks of neurodegenerative diseases (NDs).

In an epidemiological survey, the propensity of AD was found to be higher among European and American populations compared to Asia. The dietary pattern differences between Eastern and Western cultures may be among the most important reasons for the development of neurodegenerative disorders [1,12]. This was supported by the findings of Dodge et al. [13], who reported increased cases of AD in Japan after switching to a Western diet pattern. Most Asian countries consume plant-derived foods rich in antioxidants (grains, vegetables, beans, fruits, spices, herbs) as the main ingredients, followed by the consumption of seafood, poultry, and dairy products in moderation. Plants are a rich source of bioactive compounds and display vast therapeutic properties ranging from antioxidative, anti-inflammatory, cardioprotective, nephron-protective, antidiabetic, antihypertensive, antimicrobial, etc. [14,15,16]. They have also been reported to exert neuroprotective properties by affecting multiple signaling pathways [17].

Traditionally used Indian spices are a valuable collection of phytocompounds. A spice is the dried seed, fruit, root, or bark of a plant, primarily used for seasoning. As per Ayurveda, the Indian traditional system of medicine, the addition of spices not only enhances food taste but also “Agni” (digestive fire) and therefore helps in proper digestion. As cataloged by the Spice Board of India, important spices come from families such as Zingiberaceae (cardamom, turmeric), Solanaceae (chili), Fabaceae (fenugreek), Piperaceae (pepper), Myrtaceae (clove), Myristicaceae (nutmeg, mace), and Lauraceae (cinnamon). However, most spices used in Indian cuisine belong to the Apiaceae family (ajowan, asafoetida, cumin, coriander, caraway, dill, fennel).

Thus, considering their importance, Apiaceae family spices were studied to endorse the claims made in the complementary traditional system of medicine, with the main emphasis on the neuroprotective mechanism they display. This review is designed to discuss the most significant pathophysiological events linked with AD and enlists the available literature (PubMed, Google Scholar, Scopus databases) on in vivo, in vitro, and in silico studies demonstrating the pharmacological action and mechanism of important phytochemicals present in spices belonging to the Apiaceae family. Due to the presence of numerous bioactive constituents in the plants, they display a multitarget approach in ameliorating the disease symptoms, which may achieve more favorable clinical results, owing to complex AD etiology. Hence, this review can be a base for potential future complementary treatment in complex neurodegenerative diseases.

## 2. Traditional Spices and Their Neuroprotective Effect

**Figure 1:**
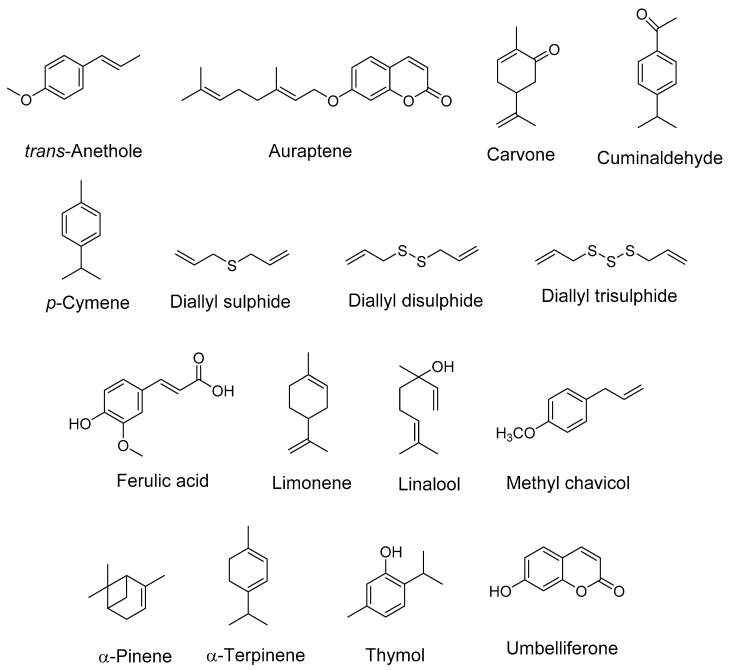
Structures of some important bioactive compounds present in spices (Apiaceae family).

**Figure 2:**
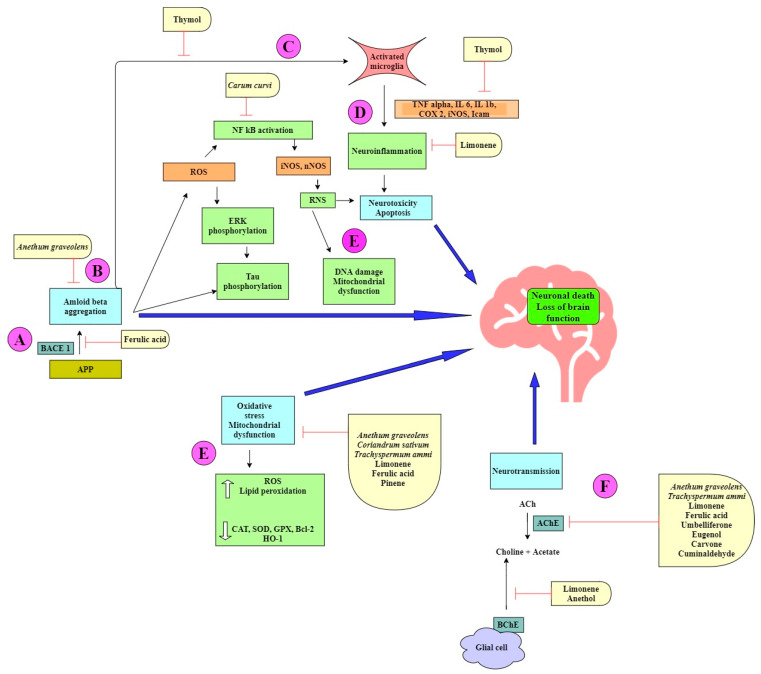
Neuroprotective mechanisms in Alzheimer’s disease displayed by spice extracts and/or natural products belonging to the Apiaceae family.

The term “spice” is derived from the Latin word “Species aromatacea”, meaning aromatic species. Spices are dried, aromatic, or pungent edible plant parts (fruit, leaves, seed, root, bark, flower), whose primary purpose in food is seasoning rather than nutrition. The bioactive compounds present in spices such as alkaloids, phenols, terpenes, and flavonoids are responsible for their therapeutic potential. Family Apiaceae (also known as Umbelliferae) has mostly aromatic flowering plants. Numerous species of this family are reported to be rich in essential and vegetable oils and hence are used in the pharmaceutical, cosmetic, perfume, and food industries [18,19]. Reports suggest the role of terpenoids and phenylpropanoids in inhibiting acetylcholinesterase (AChE) and butyrylcholinesterase (BChE) activity to a variable extent [20], which makes it important that cholinesterase inhibition by essential oils is elucidated more precisely with other chemical components. The vegetable oil obtained from umbelliferous seeds is a very rich source of a rare fatty acid, petroselinic acid (an isomer of oleic acid), and is used in chemical industries [21]. It has already been reported that fatty acids can cross the blood–brain barrier (BBB) via simple diffusion. Additionally, many transport proteins such as fatty acid binding protein 5 (FABP-5), fatty acid transport proteins-1 (FATP-1), FATP-4, and fatty acid translocase (CD36) also assist in the transport. In AD, decreased transport of many fatty acids (linoleic acid, myristic acid, palmitic acid, etc.) has been reported [22]. Moreover, expression of CD36, which is also a microglial receptor involved in the removal of Aβ, is downregulated in AD [23]. The neuroprotective mechanism of the spice active constituents and extracts belonging to the Apiaceae family is summarized in Table 1 and Table 2. The structures of important bioactive compounds from this family (Figure 1) and a diagrammatic representation for the multitarget approach by the spice extracts and phytocompounds are also depicted (Figure 2). The mechanistic aspects of Apiaceae family spices in ameliorating Alzheimer’s disease are described below.

The spice extracts and their phytochemicals exert a multitarget approach to ameliorate symptoms of AD (Figure 2). Some components prevent amyloid-*beta* aggregation by inhibiting the cleavage of the amyloid precursor protein (APP) by β-secretase (BACE-I) (Figure 2A). This causes a shift in the nonamyloidogenic pathway and reduces the levels of Aβ produced [24,53]. Aβ can self-aggregate to form oligomers and eventually amyloid plaques (Figure 2B). Some bioactive components are able to inhibit the formation of amyloid plaques by binding to Aβ, inhibiting aggregation, and thereby promoting the formation of nontoxic oligomers [45,61]. Toxic Aβ monomers and oligomers have been shown to induce microglial activation and proliferation (Figure 2C). Activated microglia secrete proinflammatory cytokines such as IL-1β and IL-6. Some natural products have been shown to reduce the levels of these cytokines [37,39,54]. Microglia also play a role in generating reactive nitrogen species (RNS), which further contribute to neurodegeneration [42,43] (Figure 2D). ROS and RNS irreversibly oxidize DNA and are important mediators of Aβ-induced neuronal cell death in the development of AD (Figure 2E). Many phytochemicals reduce oxidative stress by increasing the levels of antioxidant enzymes and reducing lipid peroxidation [30,36,37,38,39]. Acetylcholine (ACh), a neurotransmitter essential for processing memory and learning, is decreased in both concentration and function in AD (Figure 2F). Decreased levels of ACh can be restored by anticholinesterase activity of various bioactive compounds [24,25,44,63].

### 2.1. Anethum graveolens

*Anethum graveolens* (dill) seeds are generally used as a spice, flavoring, and seasoning agent in food such as pickles, salads, etc. Dill essence is rich in flavonoids, a subclass of phytoestrogens that may be accountable for having positive effects on memory enhancement, increasing levels of acetylcholine (Mesripour et al. 2016), and displaying potent antioxidant activity [64]. The most abundant constituents of essential oils in seeds were found to be carvotanacetone (21.76  ±  1.62%), dill apiole (18.65  ±  1.89%), limonene (9.01  ±  1.11%), dill ether (9.13  ±  1.12%), 4-isopropyltoluene (8.24  ±  0.89%), and myrcene (7.44  ±  0.68%) [64].

The methanolic extract of *A. graveolens* seeds demonstrated moderate neuroprotective effects in PC12 cells treated with Aβ (25–35) aggregates with an ED50 value of 18.8 µg/mL [45]. The administration of *A. graveolens* ethanolic leaves extract significantly improved the learning and memory damage induced by scopolamine in Morris water maze and elevated plus maze experiments. A substantial decrease in AChE activity, increased activity of brain antioxidant enzymes such as superoxide dismutase, and decreased lipid peroxidation were also observed in the study. The most potent action was seen at a dose of 400 mg/Kg body weight [46].

The memory-enhancing activity of *A. graveolens* aqueous extract (100, 200, and 300 mg/Kg body weight) was also appraised by the conditioned avoidance response (CAR) technique in rats using Cook’s pole climbing apparatus [48]. Changes in cognition, retention, and recovery in rats were dose dependent. The extract also inhibited lipid peroxidation in both liver and brain tissues, suggesting the role of extract in reducing oxidative stress. In another study, the protective effect of *A. graveolens* aqueous extract was studied on hypercholesterolemia-induced cognitive deficits (HCDs) and oxidative stress in hippocampus tissues of rats [47,49]. HCD considerably augmented serum cholesterol levels, induced Aβ deposition, transformed morphology of hippocampus, and impaired memory function. However, the changes were reversed by administration of *A. graveolens* extract, which acted by increasing antioxidant levels in the brain, lowering serum cholesterol, retarding Aβ deposition, and normalizing hippocampal morphology.

PM52, a combined extract of *Cissampelos pareira* and *A. graveolens*, was evaluated against age-related cognitive impairment in a rat model. The data proposed that the cognitive-enhancing effect of PM52 might be due to suppression of AchE, resulting in increased levels of acetylcholine, a neurotransmitter that plays an important role in learning and memory and enriching neuron density in hippocampus by reducing the oxidative stress [50].

Hence, *A. graveolens* extracts and active constituents improved cognitive function in AD brain mainly by inhibiting AChE, improving oxidative stress conditions, and retarding amyloid β aggregation.

### 2.2. Carum carvi

*Carum carvi* (caraway) is a biennial herb, the dried fruit of which is used as a spice due to its pleasing odor and sharp taste. The main components of essential oil are carvone (44.5–95.9%) and limonene (1.5–51.3%) and minor amounts of β-myrcene, *trans*-dihydrocarvone, *trans*-carveole (0–0.2%), α-pinene, sabinene, *n*-octanal, *trans*-β-ocimene, δ-terpinene, linalool, *cis*- and *trans*-limonene oxide, *cis*-dihydrocarvone, *cis*-carveol, perillaldehyde, *trans*-anethole, and *trans*-β-caryophyllene [65].

Microglia plays a dual role (neuroprotective or neurotoxic) in the progression of AD [66]. Microglia are regarded as initiators of neuroinflammation [67] and might play a role in the atypical networking in AD brain [68]. Various proinflammatory and neurotoxic substances, such as NO, iNOS, and COX-2, are produced by activated microglia. Therefore, reducing neuroinflammation by reducing microglia activation could be a promising therapeutic target in treatment of neuroinflammatory-mediated neurodegenerative diseases such as AD. It has already been reported that natural products with antineuroinflammatory activity are able to produce antiamyloid [69]. In an in vitro study, *C. carvi* aqueous extract was evaluated for its protective effects on LPS-activated neuroinflammation in BV-2 microglial cells. The extract inhibited LPS-induced phosphorylation/degradation of IκBα and translocation of NF-κB/p65 subunit in a concentration-dependent manner, which means the *C. carvi* extract plays an important role in regulating NF-κB signaling [51]. Carvone, the major component of *C. carvi* oil, is known to exhibit anti-inflammatory properties by inhibiting the synthesis of leukotrienes and prostaglandins [26]. Therefore, it is quite possible that carvone plays a role in modulation of the NF-κB pathway. Antioxidant, adaptogenic, and memory-enhancer activities of *C. carvi* aqueous extract were evaluated in rats using Cook’s pole climbing apparatus. The extract decreased lipid peroxidation in liver and brain homogenates [52]. Essential oil (EO) of *C. carvi* displayed strong in vitro anti-AChE activity (IC_50_ = 0.82 ± 0.05 mg/mL) compared to the reference drug galantamine (IC_50_ = 1.05 ± 0.05 mg/mL) [53]. Pharmacokinetics profiling of selected components of essential oils indicated their ability to penetrate the blood–brain barrier moderately (log BB values = 0.818–0.478). They also demonstrated high CaCO-2 permeability (log Papp values > 0.90 cm/s). All tested compounds were predicted neither substrates nor inhibitors of the human cytochrome P450 (CYP) isoforms. The boiled-egg graph (WLOGP vs. TPSA) prediction of GI absorption and BBB permeation, which helps in the calculation of polarity and lipophilicity, indicated that they possess a high probability of brain penetration [53].

Carvone, the main constituent of *C. carvi*, has been reported as an AChE inhibitor (IC_50_ = 2.9 ± 0.12 mM) compared to the reference drug galantamine (IC_50_ = 0.14 ± 0.005 mM) [27]. The inhibition is noncompetitive [70]. Additionally, docking studies revealed a putative H-bond interaction between the carvone and Tyr337 (2.92Å) of AChE, creating an anionic subsite. Other binding sites include Trp86, Tyr133, Tyr337, Phe338 (an anionic subsite), His447, Ser203 (an esteratic site), Gly121, Gly122 (oxyanion hole), Ile451, Gly448, Glu202, Gly120, and Ser125, all of which are most important portions of the AchE binding site [27]. In a recent study, (‒)-*cis*-carveol, a reduction product of carvone, improved Aβ_1-42_-induced memory deficits in an animal model, examined by Y-maze and radial arm maze in vivo tests [71]. The biochemical analyses of the hippocampus homogenates showed a reduction in oxidative stress parameters caused by Aβ_1-42_, suggesting the role of carveol in neuroprotection.

The second most important constituent in *C. carvi* is limonene, a monoterpene. Its protective role in spatial memory and anxiety has been established in a rat model exposed to immobilized stress [72]. Limonene also had a positive effect on scopolamine-induced amnesia, where it improved the modifications caused by scopolamine in a short-term memory test. It ameliorated the levels of oxidative stress markers (MDA, SOD, GSH) and inhibited AchE and BchE [20,37]. A study examined the effectiveness of limonene against Aβ_1-42_-induced neurotoxicity in a *Drosophila* model of AD. The results showed that limonene suppressed the neuronal cell death induced by Aβ_42_ and reduced oxidative stress, which prevented ERK phosphorylation [39]. In another study, the neuroprotective effect of limonene against neurotoxicity elicited by Aβ_1-42_ in Hoechst 33,258 cell lines was observed. Limonene decreased ROS production and prevented the upregulation of Kv3.4 (voltage-gated potassium channel) activity at 10 µg/mL. This channel is overexpressed in AD and other neurodegenerative diseases [36]. Downregulation of Kv3.4 expression by limonene prevented cell death in primary cortical neurons, thus confirming its neuroprotective function in AD [38]. Moreover, limonene displayed a specific activity almost comparable to galantamine, the reference drug used against AchE.

Hence, maintaining the levels of antioxidant enzymes, inhibiting AchE, suppressing neuroinflammation and downregulating voltage-gated potassium channels are the main roles of *C. carvi* extract and active components in combating AD.

### 2.3. Coriandrum sativum

*Coriandrum* (coriander) is a feathery annual plant, used as both an herb and a spice. The main constituents of coriander oil are linalool (64.2−79.9%), γ-terpinene (5.8−13.6%), neryl acetate (2.3−8.4%), α-pinene (2.8−7.1%), and *p*-cymene (1.1−3.6%) [73]. In the Aβ_1-42_ AD model, a test group of rats as made to inhale essential oil (1% and 3%) from *C. sativum* seed. Inhalation of essential oil considerably reduced levels of LDH and MDA, with an increase in glutathione peroxidase levels in the hippocampal region of rats. Additionally, there were fewer amyloid deposits in rats treated with EO. Specifically, linalool was found to be the active constituent in the EO; therefore, it can be speculated that linalool is responsible for cognitive-enhancing effects, along with antiapoptotic activities in Aβ_1-42_-treated rats. The antioxidant defense, along with a decrease in lipid peroxidation, could be correlated with involvement of linalool in neuroprotection [40]. *C. sativum* seed extract is reported to be nontoxic in up to 3000 mg/kg body weight (BW) and, thus, can be considered safe for intake [74]. In a recent experiment, *C. sativum* seed extract (200 mg/Kg BW) improved memory impairment in senescence-accelerated mouse-prone 8 (SAMP8) mice. The mRNA levels of nNOS were higher in diseased the frontal lobe of diseased animals, which decreased significantly with the treatment of extract, suggesting that *C. sativum* can reduce the production of RNS and ROS and thus improve oxidative stress conditions. Moreover, the mRNA level of neurofilament light (NF-L), an important protein in memory retention and synaptic plasticity, was found lower in the frontal lobe and hippocampus of untreated mice, indicating neuronal damage. However, the mRNA levels NF-L were elevated after extract administration [54], indicating the role of *C. sativum* in neuroprotection. Previous studies have shown that α-pinene, γ-terpinene, and many monoterpenoids have anti-AchE activity [27,75,76]. As these phytocompounds are present in *C. sativum*, it is very much likely that its extract will also show anti-AchE activity.

Therefore, as described above, *C. sativum* extract and its bioactive compounds play an imperative role in neuroprotection by reducing ROS/RNS, elevating the level of an important protein involved in synaptic plasticity, and suppressing AchE activity.

### 2.4. Cuminum cyminum

*Cuminum cyminum* (cumin) is the second most popular spice in the world after black pepper. It is a slender, annual, glabrous herb, and its seeds have been traditionally used in colic pain, abdominal discomfort, and deficient lactation. It also possesses antioxidant, anti-inflammatory, antibacterial, and antidiabetic activities [77,78,79]. Cumin seed is valued for its aroma, which is due to the presence of cuminaldehyde, cuminic alcohol, *p*-cymene, *o*-cymene, γ-terpinene, α-terpinene, *p*-menthadienol, and β-pinene as some of its chief components [80,81]. High levels of a rare, monounsaturated, omega-12 fatty acid, petroselinic fatty acid (C18:1), are also present in cumin [82].

Cuminaldehyde was displayed to play a role in neuroprotection and spatial learning and memory enhancement through the modulation of genes (Bdnf, Icam, ApoE, IL-6) coding for neurotrophic factors and/or those associated in damaging synaptic plasticity in both in vitro and in vivo experiments [28]. Memory-enhancing activity of *C. cyminum* water extract was reported in animal models of AD by maintaining the levels of antioxidant enzymes [55,56]. It also showed competitive inhibitory activity for AchE in vitro at low concentrations (12.5 μg/mL and 25 μg/mL) and mixed inhibition at higher concentrations (50 µg/mL) [57,83]. Additionally, cumin essential oil (cuminaldehyde) and its *n*-hexane fraction strongly inhibited α-synuclein (α-SN) aggregation in a concentration-dependent manner in PC12 cells by inhibiting the fibrillation process [59]. In a docking study [84], cuminaldehyde showed a docking score of −7.5, similar to that of selegiline (a known inhibitor), against monoamine oxidase (MAO-B), an enzyme that deactivates neurotransmitters such as dopamine, and other neuromodulatory amines such as polyamines [85]. The expression of MAO-B is enhanced in the hippocampus and cerebral cortex of AD brains compared to healthy brains [86]. Therefore, MAO-B inhibitors can be an alternative, Aβ-independent strategy to target AD [87].

To summarize, *C. cyminum* extract and important phytocompounds play a role in neuroprotection and cognitive enhancement by modulating the genes involved in preserving synaptic plasticity, repressing oxidative stress, and inhibiting enzymes involved in neurotransmission.

### 2.5. Ferula asafoetida

*Ferula asafoetida* (asafoetida) is a gum-like exudate from underground rhizomes or taproots of the plant. Asafoetida usually contains approximately 40–64% resin, 25% endogenous gum, and 10–17% volatile oil. The resin portion mainly contains asaresinotannols A and B, ferulic acid, and umbelliferone. The volatile oil is rich in various organosulfide compounds, such as 2-butyl-propenyl-disulfide, diallyl sulfide, diallyl disulfide, and dimethyl trisulfide [88,89]. The organosulfides are largely accountable for the peculiar smell and flavor of asafoetida.

In a previous report, in vitro and in vivo findings indicated that two natural components of some *Ferula* species, namely umbelliferone and ferulic acid, act as competitive AchE inhibitors. Umbelliferone markedly replenished nuclear factor erythroid-derived 2-like 2 (Nrf2) and heme oxygenase-1 (HO-1) levels in streptozotocin (STZ)-induced cognitive dysfunction in rats [44]. Oral administration of ferulic acid (30 mg/Kg) for 6 months decreased cleavage of the β-carboxyl-terminal APP fragment, BACE-I activity, neuroinflammation, and stabilized oxidative stress in a transgenic PS/APP mouse model of AD. As a result, significant cognitive improvement was observed in the animals [32]. It also inhibits BACE1 enzymatic action, as well as its mRNA expression level [90]. In addition, eugenol and limonene, which are among volatile constituents of some *Ferula* species, were shown to inhibit AchE [91]. Moreover, auraptene, which is a prenylated coumarin present in some *Ferula* species, displayed a neuroprotective effect with mild AchE-inhibitory activity [25]. Long-term administration of ferulic acid, another important component of *Ferula*, was reported to protect mice against Aβ-induced learning and memory deficits in vivo [31,35] and protect neurons against Aβ-induced oxidative stress and neurotoxicity in vitro [34] by modulating oxidative stress directly and by inducing protective genes such as heme oxygenase-1 (HO-1) and heat shock protein 72 (Hsp72). In another study, the same compound showed a protective role in trimethyltin-induced memory injury in a mouse model and decreased AchE activity [92].

Ferulic acid interferes with the apoptotic pathways induced by oxidative stress and inflammation due to Aβ aggregation [33]. Ferulic acid is also a potent antioxidant and has anti-inflammatory properties. Most importantly, it directly alters the kinetics of Aβ fibril formation and can directly inhibit plaque formation in vitro [93]. However, the oral administration of ferulic acid was unable to display any significant effect on Aβ oligomers or Aβ deposition in vivo [94], which could be due to poor absorption or difficulty in crossing BBB in the body. Ferulic acid has also been involved in enhancing the cell stress response by regulating heme oxygenase/Hsp70 [95], heme oxygenase/biliverdin reductase (HO/BVR) system [96,97], superoxide dismutase (SOD), catalase [98], ERK ½, and Akt [99] and inhibiting caspases [100].

Diallyl sulfide (DAS), diallyl disulfide (DADS), and diallyl trisulfide (DATS) are lipid soluble allyl sulfur compounds [101] and have potent antioxidant activity. They have the ability to trap trichloromethyl and trichloromethyl peroxyl free radicals. DADS also inhibited carbon tetrachloride-induced lipid peroxidation [30]. As lipid peroxidation increases in AD, these compounds might display antioxidant potential and reduce oxidative stress in the brain.

Thus, modulating oxidative stress, suppressing neuroinflammation, and inhibiting BACE-I and AchE activity are the modes of action of *Ferula asafoetida* in targeting AD.

### 2.6. Foeniculum vulgare

*Foeniculum vulgare* (fennel) is a perennial herb with seeds rich in essential oil, responsible for its aroma and taste. GC-MS analysis identified the main components of oil as *trans*-anethol (84.1–86.1%), fenchone (7.13–8.86%), limonene (3.0–3.3%), and methyl chavicol (2.5–2.7%) [102]. Fennel seeds also contain fatty acids such as petroselinic acid (62.08–66.71%), 10-nonadecanone (4.70–22.80%), and linoleic acid (1.32–7.59%) and minor amounts of oleic acid, stearic acid, eicosanoic acid, and linolenic acid [103].

In a scopolamine-induced rat model, fennel water extract potently inhibited lipid peroxidation in both rat liver and brain homogenates and improved memory, which can be correlated with the strong, antioxidant capacity of the extract [60]. Moreover, methanolic extract also ameliorated symptoms in a scopolamine-induced mouse model [104]. In a similar experiment, ethanolic extract (200 mg/Kg/d) displayed a neuroprotective effect in a lead-induced neurotoxicity mouse model by restoring the levels of oxidative stress markers and APP isoforms in cortex and hippocampus [61].

More recently, both fennel and trans-anethole were reported to prevent and treat stress-induced neurological disorders, such as memory and learning in isolated rats [105]. by strengthening the antioxidant system and reducing neuronal inflammation. Anethole also possesses both AchE and BchE inhibitory activity with IC_50_ values of 39.89 ± 0.32 μg/mL and 75.35 ± 1.47 μg/mL, respectively [24]. Methyl chavicol is a phenylpropene or estragole in nature, and these compounds are potent AchE inhibitors [106] with an IC_50_ of 0.337 µM [41]. Another compound, limonene (+), displayed a neuroprotective effect in Aβ1-42-induced neurotoxicity in a Drosophila model of AD by reducing ROS production, kinase phosphorylation, neuroinflammation, and cell death without affecting Aβ42 accumulation and aggregation [107].

Limonene also had a beneficial effect on scopolamine-induced amnesia by reducing oxidative stress markers and by inhibiting AchE and BchE activity (24.97% and 69.12%, respectively) [37]. The interaction of limonene with the hydrophobic choline esterase binding site could be the reason for inhibition [108].

In summary, *F. vulgare* extract and its bioactive components improve AD condition by inhibiting the enzymes involved in neurotransmission, suppressing oxidative stress, and neuroinflammation.

### 2.7. Trachyspermum ammi

*Trachyspermum ammi* (also known as ajowan, thymol seeds, bishop’s weed, or carom) is an annual herbaceous and aromatic plant. It primarily contains essential oils such as thymol, α- and γ-terpinene, *p*-cymene, and α- and β-pinenes [109].

In a recent experiment, alprazolam, scopolamine, and electroshock were used to induce amnesia in mice, and the effect of T. ammi seed (0.5%, 1.0%, and 2.0% *w*/*w* of normal diet) was studied on the learning and memory of mice using the passive avoidance paradigm and object recognition task (ORT) [62]. The supplementation of *T. ammi* not only enhanced step-down latency passive avoidance response and discrimination index of ORT animals but also considerably reduced brain AchE activity and oxidative damage by decreasing the levels of MDA and nitrite and increasing GSH. In a scopolamine-induced zebrafish model of memory impairments, T. vulgaris essential oil was reported to augment cognitive function through action on cholinergic neurons [63]. The essential oil decreased AChE activity and increased brain antioxidant capacity. The chief components detected by GC-MS were thymol (42%) and *p*-cymene (19%), which indicates the role of these two in neuroprotection. The activity of p-cymene (50 and 100 mg/Kg) was also assessed in a Aβ_1–42_-treated rat model of AD [29]. Results demonstrate that both doses have a positive effect on the learning and memory functions of the rats and reduce amyloid plaque deposition. Thymol possesses strong, antioxidant properties and inhibits β-amyloid in (Aβ)-induced cognitive-impaired rats [42,43], which suggests that thymol can attain the appropriate concentrations required to exert its therapeutic effects on neurons by crossing the BBB. Thymol treatment significantly decreased the number of activated astrocytes and microglia in the striatum region in ROT-injected animals. The reduction in the number of glial cells and reduced expression of COX-2 and iNOS following thymol treatment is suggestive of its anti-inflammatory effects [110].

In a study, α-pinene improved learning and memory in scopolamine-induced memory deficit in C57BL/6 mice by ameliorating the expression of proteins related to the synthesis of acetylcholine and antioxidant defense system [111]. However, α-terpinene displayed its neuroprotective action by altering the activity of enzymes responsible for neuronal plasticity and hydrolysis of ADP and ATP in female Wistar rats [112].

To conclude, *T. ammi* extract and active constituents act by inhibiting AChE, reducing amyloid aggregation, preventing neuroinflammation, and enhancing brain antioxidant activity.

## 3. Clinical Studies

A number of clinical trials have been conducted to study the effect of spices belonging to the Apiaceae family in several diseases such as diabetes [113,114], obesity [115,116,117], hyperlipidemia [118,119], metabolic syndrome [120,121], functional dyspepsia [122], neuropathic pain [123], arthritis [124], skin diseases [125], gynecological problems [126,127,128,129,130], and dental diseases [131]. All these studies ruled out any safety concerns with these spices. However, no preclinical or clinical study has been reported for the effectiveness of these spices in neurodegenerative diseases, even though in vivo and in vitro literature suggest their immense neuroprotective potential. Hence, there is a tremendous scope of preclinical and clinical trials on these spices.

## 4. Future Perspectives

The promising pharmacological effects of various plant extracts from the identified Apiaceae spices may pave the way for a deeper investigation of potential biologically active natural products. Aside from allyl sulfides, asaresinotannols A and B, umbelliferone, and ferulic acid, which were characterized in *Ferula asafoetida*, the reported components from the Indian spices were mostly monoterpenes concentrated in the essential oils. Over the years, the isolation of natural products possessing diverse structures and pharmacological activities was made possible using their crude extracts. Depending on the nature of the research, several approaches can be utilized in examining the potential active metabolites. These natural products have been used for further medicinal chemistry research, paving the way for compounds in the pipeline for clinical studies. The limited information on the potential bioactive natural products from the spices of the Apiaceae family has disclosed the need for in-depth phytochemical and pharmacological analyses. Currently, bioassay-guided isolation is our laboratory’s custom in the identification of neuroprotective natural products from plant sources. Aside from the essential oils, our interest is focused on examining neuroprotective compounds from the crude extracts, utilizing both chromatography and well-established bioassays in our laboratory.

## 5. Conclusions

AD is a predominant neurodegenerative disease with no effective drug treatment to date; therefore, the investigation of new phytochemicals from plant sources is utterly required. Plant secondary metabolites such as alkaloids, flavonoids, and phenolic acids play a key role in ameliorating disease conditions in AD. Essential oils also play an important role in AD pathogenesis, as they are a rich source of antioxidants, and most of them exhibit cholinesterase inhibitory potential. It is understood that as plant extracts contain several bioactive compounds, they can work additively or synergistically to display a variety of neuroprotective mechanisms that might be an effective tactic in AD drug discovery. Further research on ideal dosage, mode of action at both the molecular and cellular levels, in vivo effects, and pharmacokinetic profile of the discussed phytocompounds and plant extracts can lead to more effective new anti-AD product development in the future.

## Figures and Tables

**Table 1:** List of biologically active compounds identified from the Apiaceae species.

Natural Product	Identification in Apiaceae Species	Reported Mechanism Associated with Alzheimer’s Disease	Reference
*trans*-Anethole	*Foeniculum vulgare*	AChE-inhibitory activity BChE Inhibitory activity	[24]
Auraptene	*Ferula* sp.	AChE-inhibitory activity	[25]
Carvone	*Carum carvi*	Neuroinflammatory effects by inhibiting leukotrienes and prostaglandins and modulation of NF-ΚB signaling pathway AChE-inhibitory activity	[26,27]
Cuminaldehyde	*Cuminum cyminum*	Spatial learning and memory enhancement Modulation of BDNF, Icam, ApoE, and IL-6 genes	[28]
*p*-Cymene	*Coriandrum sativum* *Trachyspermum ammi* *Cuminum cyminum*	Improved learning and memory functions Reduced the deposition of amyloid plaques	[29]
Diallyl sulfide Diallyl disulfide Diallyl trisulfide	*Ferula asafoetida*	Antioxidant activity by trapping trichloromethyl and trichloromethyl peroxyl free radicals Inhibition of CCl_4_-induced lipid peroxidation by diallyl disulfide	[30]
Ferulic acid	*Ferula asafoetida*	AChE-inhibitory activity In vivo cognitive improvement by inhibiting BACE-1, decreasing cleavage of C-terminal APP fragment, neuroinflammatory activity, and stabilization of oxidative stress Enhancement of learning and memory deficits by inhibiting Aβ plaques in vivo Inhibition of Aβ fibrillization and aggregation	[31,32,33,34,35]
Limonene	*Anethus graveolens* *Carum carvi* *Foeniculum vulgare*	Increased the levels of oxidative markers MDA, SOD, and GSH AChE-inhibitory activity BChE inhibitory activity Suppression of Aβ42-induced cell neurotoxicity Reduction in ROS levels Downregulation of the neurotransmitter Kv3.4 expression Reduction of kinase phosphorylation Neuroinflammatory effects	[36,37,38,39]
Linalool	*Coriandrum sativum* *Carum carvi*	Reduction of lipid peroxidation Cognitive enhancement Antiapoptosis in Aβ_42_-treated rats	[40]
Methyl chavicol	*Foeniculum vulgare*	AChE-inhibitory activity	[41]
α-Pinene	*Trachyspermum ammi* *Coriandrum sativum* *Carum carvi*	Improved learning and memory functions by inhibiting AChE and oxidative stressors	[27]
α-Terpinene	*Carum carvi* *Coriander sativum* *Cuminum cyminum* *Trachyspermum ammi*	AChE-inhibitory activity Inhibition of enzymes responsible for neuronal plasticity and hydrolysis of ADP and ATP	[27]
Thymol	*Trachyspermum ammi*	Antioxidant activity Inhibition of Aβ plaques in cognitive-impaired rats Neuroinflammatory effects by reduction of the activated astrocytes and microglia and downregulation of COX-2 and iNOS expression in vivo	[42,43]
Umbelliferone	*Ferula asafoetida*	AChE-inhibitory activity Increased the expression of Nrf2 and heme oxygenase-1 (HO-1) in vivo	[44]

**Table 2:** Neuroprotective activities in Alzheimer’s disease displayed by Apiaceae family spice extracts.

Plant Species	Extract	Reported Activity Associated with Alzheimer’s Disease	Reference
*Anethum graveolens*	Methanolic seed extract	Neuroprotective effects in Aβ-induced PC12 cells	[45]
	Ethanolic leaf extract	Enhancement of learning and memory in vivo AChE-inhibitory activity Amelioration of antioxidant SOD enzyme Reduction of lipid peroxidation	[46]
	Aqueous extract	Improvement of memory impairment in vivo by reducing oxidative stress In vivo lowering of serum cholesterol, inhibition of Aβ deposition, and normalization hippocampal morphology	[47,48,49]
	PM52 extract (combined extract of *A. graveolens* and *Cissampelos pareira*)	In vivo Cognitive enhancement by suppressing AChE and reducing levels of ROS	[50]
*Carum carvi*	Aqueous seed extract	Neuroinflammatory activity by regulating the NF-kB signaling pathway In vivo antioxidant (reduction of lipid peroxidation), adaptogenic, and memory enhancement activities	[51,52]
	Essential oil	Inhibition of AChE activity	[53]
*Coriandrum sativum*	Seed extract	Improvement of memory impairment by increasing the level of mRNA NF-L and decreasing the mRNA nNOS	[54]
*Cuminum cyminum*	Aqueous extract	In vivo memory enhancement, antioxidant activity, and inhibition of AChE	[55,56,57,58]
	Hexane extract and cumin essential oil	Inhibition of α-synuclein aggregation in PC12 cells	[59]
*Foeniculum vulgare*	Aqueous extract	In vivo inhibition of lipid peroxidation and antioxidant activity	[60]
	Ethanolic extract	In vivo neuroprotective effects in lead-induced neurotoxicity by decreasing the levels of oxidative stress and APP isoforms	[61]
*Trachyspermum ammi*	Seed extract	In vivo learning and memory enhancement by reducing the brain AChE activity, and prevention of oxidative damage by decreasing the levels of MDA and nitrite and increasing GSH	[62]
	Essential oil	AChE-inhibitory activity In vivo increased in brain antioxidant capacity	[63]
